# Complementary and alternative medicine for patients with chronic fatigue syndrome: A systematic review

**DOI:** 10.1186/1472-6882-11-87

**Published:** 2011-10-07

**Authors:** Terje Alraek, Myeong Soo Lee, Tae-Young Choi, Huijuan Cao, Jianping Liu

**Affiliations:** 1 National Research Center for Complementary and Alternative Medicine, University of Tromsø, Norway; 2 Brain Disease Research Center, Korea Institute of Oriental Medicine, Daejeon, South Korea; 3 Center for Evidence-Based Chinese Medicine, Beijing University of Chinese Medicine, Beijing, China

## Abstract

**Background:**

Throughout the world, patients with chronic diseases/illnesses use complementary and alternative medicines (CAM). The use of CAM is also substantial among patients with diseases/illnesses of unknown aetiology. Chronic fatigue syndrome (CFS), also termed myalgic encephalomyelitis (ME), is no exception. Hence, a systematic review of randomised controlled trials of CAM treatments in patients with CFS/ME was undertaken to summarise the existing evidence from RCTs of CAM treatments in this patient population.

**Methods:**

Seventeen data sources were searched up to 13th August 2011. All randomised controlled trials (RCTs) of any type of CAM therapy used for treating CFS were included, with the exception of acupuncture and complex herbal medicines; studies were included regardless of blinding. Controlled clinical trials, uncontrolled observational studies, and case studies were excluded.

**Results:**

A total of 26 RCTs, which included 3,273 participants, met our inclusion criteria. The CAM therapy from the RCTs included the following: mind-body medicine, distant healing, massage, tuina and tai chi, homeopathy, ginseng, and dietary supplementation. Studies of qigong, massage and tuina were demonstrated to have positive effects, whereas distant healing failed to do so. Compared with placebo, homeopathy also had insufficient evidence of symptom improvement in CFS. Seventeen studies tested supplements for CFS. Most of the supplements failed to show beneficial effects for CFS, with the exception of NADH and magnesium.

**Conclusions:**

The results of our systematic review provide limited evidence for the effectiveness of CAM therapy in relieving symptoms of CFS. However, we are not able to draw firm conclusions concerning CAM therapy for CFS due to the limited number of RCTs for each therapy, the small sample size of each study and the high risk of bias in these trials. Further rigorous RCTs that focus on promising CAM therapies are warranted.

## Background

Throughout the world, patients with chronic diseases tend to be high utilisers of health care resources and/or the health care system. Such patients are also frequent users of Complementary and Alternative Medicine (CAM) services, which are present either within or outside the National Health Service. The reasons for using CAM are diverse; however, hope, engagement in one's own health and positive expectations of treatment efficacy are nearly always present. Patients with chronic fatigue syndrome (CFS) are no exception. CFS is a challenging illness for patients, as well as those close to them, health care providers and society in general. Western medicine usually has potent treatments readily available for diseases with a single cause and a well-described pathophysiology. However, as of yet, no single cause of CFS has been discovered, although potential factors, which are still questionable, have been identified [1-3]. Several treatments for this condition have been explored; however, none has shown persistent or consistently significant outcomes in this patient population [4-6]. Although several CAM treatments for CFS patients were described in a previous review, the review only included literature up to April 2007 [7]. Furthermore, a new quality assessment tool (the Cochrane risk of bias tool) has since been proposed to enhance the validity of systematic reviews [8]. Therefore, the aim of our review was to systematically summarise and critically evaluate the data from RCTs of CAM treatment for patients with CFS.

## Methods

### Data sources

We searched the following electronic databases up to 13^th ^August 2011: Medline, PsycInfo, Alternative Medicine (AMED), the Cumulative Index to Nursing & Allied Health Literature (CINAHL), EMBASE, and the Cochrane Library 2011 (Issue 5). We also searched the Chinese databases (China Network Knowledge Infrastructure (CNKI; 1979-2010), the Chinese Scientific Journal Database VIP (1989-2010), the Wan Fang Database (1985-2010), and the Chinese Biomedicine (CBM) database (1978-2010); the Korean medical databases (including Korean Studies Information, DBPIA, Korea Institute of Science and Technology Information, Research Information Service System, KoreaMed, and National Assembly Library); and Japanese databases (Japan Science and Technology Information Aggregator, Electronic). The search strategy is listed in Additional file 1. In addition, we manually searched our own files, *Focus on Alternative and Complementary Therapies *and *Forschende Komplementärmedizin*. The references in all located articles were also searched.

### Selection Criteria

All randomised controlled trials (RCTs) of any type of CAM therapy, with the exception of acupuncture and complex Chinese herbal medicines, for the treatment of CFS were included, regardless of blinding or the published language. Cochrane reviews of trials testing acupuncture type therapies [9] are ongoing, and as a result, this topic was excluded. We included RCTs that tested a single herb for CFS. RCTs testing complex herbal medicines for CFS were excluded, as it is not possible to isolate the effects of single herbs. Trials were included if they used CAM as either the sole treatment or as an adjunct to other treatments, which occurred in cases where the control group also received the same concomitant treatments as the CAM group. Studies comparing two different forms of CAM and those in which no clinical data were reported were also excluded. Cognitive behavioural interventions were not considered to be a part of CAM and were therefore excluded. If cognitive behavioural intervention was used as a control, the trial was included. Trials that employed CAM as the sole treatment or as an adjunct to other treatments were included. Dissertations and abstracts were included if they contained sufficient detail for critical evaluation. Hard copies of all articles were obtained.

### Data Extraction, Quality, and Risk of Bias Assessment

All articles were read, and data were extracted from the articles based on predefined selection criteria by two independent reviewers (MSL and TYC). To evaluate the methodological quality of the RCTs, the risk of bias was determined using the Cochrane classification for eight criteria: random sequence generation, allocation concealment, patient blinding, assessor blinding, reporting of dropout or withdrawal, intention-to-treat analysis, selective outcome reporting and other potential biases [8].

## Results

### Study description

**Figure 1:**
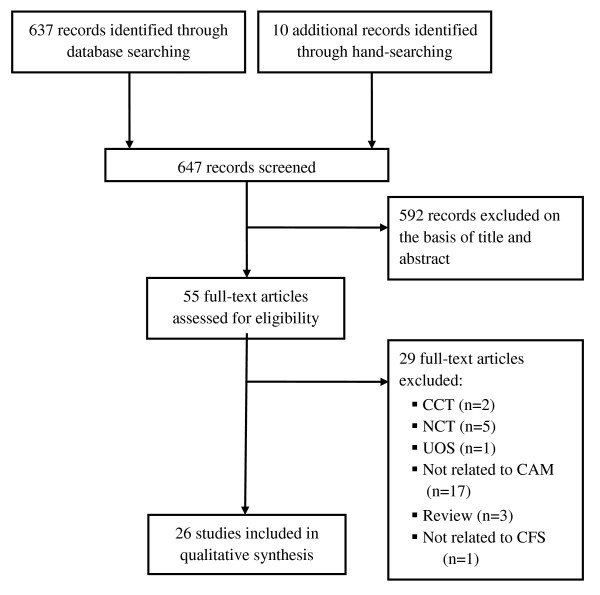
**Flow diagram of literature search**. CCT: controlled clinical trial; NCT: not clinical trial; UOS: uncontrolled observational study; CAM: complementary and alternative medicine; CFS: chronic fatigue syndrome

We screened 647 relevant articles, and 592 were excluded, leaving us with 55 full-text eligible articles. Of these, 29 more were excluded. The remaining 26 RCTs met our inclusion criteria (Figure 1).

Key data from these studies are summarised in Tables 1 and 2[10-35]. The RCTs included in the table employed the following treatments: mind-body medicine (4) [10-13], massage (2) [14,15], tuina and tai chi (1) [16], homeopathy (2) [17,18], ginseng (1) [19], nicotinamide adenine dinucleotide (NADH) (2) [20,21], and dietary supplements (14) [22-35]. A placebo procedure was employed in 16 trials [17-20,22-27,29-34]. Twenty of the included trials adopted a two-arm parallel group design [10-12,14,15,17-27,29,30,33,35], three adopted a three-arm parallel group design [16,28,34], and one used a four-arm parallel group design [13], while two trials employed a cross-over design [31,32]. Nine trials adopted the CDC criteria for the diagnosis of CFS [11,20,22,24,25,27,28,31,32], five studies diagnosed CFS according to the criteria published by Fukuda [10,15,16,19,21], five used the Oxford criteria [12,17,18,30,35], two combined with Fukuda and Oxford criteria [13,23], and one used a different classification system [33].

**Table 1:** Summary of randomised clinical studies of complementary and alternative medicine for patients with chronic fatigue syndrome

First author (year)	Sample size	Diagnosis	Intervention group (Regime)	Control group (Regime)	Main outcomes	Intergroup differences	Adverse events
	Gender (M/F)						
	Mean age (range)						
Clollinge (1998) [10]	70	Fukuda	(A) Qigong (2 hr weekly for 9 weeks, n = 37) plus mindfulness meditation and group discussion	(B) No treatment (n = 33)	SF-36 Heath transition score	No between group analysis	n.r.
	(10/50)						
	27-61						
Dybwad (2007) [11]	31 (4/27)	CDC	(A) Qigong (2 hr weekly for 12 weeks), plus meditation, n = 15)	(B) No treatment (n = 16)	1) Work capacity (VO_2max_)	1) P = 0.01	n.r.
	17-62				2) Fatigue severity	2) P < 0.05	
					3) SF36	3) NS	
Surawy (2005) [12]	18	Oxford	(A) MBSR (once weekly for 8 weeks, n = 8)	(B) Wait-list (once weekly for 8 weeks, n = 9)	1) HADS Anxiety	1) P = 0.00	n.r.
	8-Oct				2) HADS Depression	2) NS	
	18~65				3) Chalder Fatigue Scale	3) NS	
	(n.r)				4) SF36 Physical Functioning	4) NS	
Walach (2008) [13]	409	Fukuda or Oxford	(A) Distant healing (blinded, n = 105)	(C) No distant healing (blinded, n = 95)	SF-36	NS for both the mental and physical component	n.r.
			(B) Distant healing (not blinded, n = 102)	(D) No distant healing (not blinded, n = 109)			
Field (1997) [14]	20	Not stated	(A) Massage therapy (twice weekly for 5 weeks, n = 10)	(B) Sham TENS (twice weekly for 5 weeks, n = 10)	1) CESD	1) P < 0.005	n.r.
	16-Apr				2) Fatigue	2) P < 0.05	
	n.r.				3) Pain	3) P < 0.005	
	-47				4) Sleep	4) P < 0.05	
Wang (2009) [15]	182	Fukuda	(A) Intelligent-turtle massage (5 times weekly, 10 times as a course, for 2 courses with a one-week interval in between, n = 91)	(B) Massage(45 min, 5 times a week, 10 times as a course, n = 91)	Physical Symptoms	P < 0.05	n.r.
	141/40						
	21-62						
	(n.r)						
Liu (2010) [16]	90 (44/46)	Fukuda	(A) Tuina (once 2 days for 15 times, n = 30)	(C) Fluoxetine (20 mg/d, 1 month, n = 30)	Total effective rate	A vs. B, NS; A vs. C, P < 0.05; B vs. C, NS	(C); 17 insomnia dizziness vexation nausea, hypodynamia
	26.4~46.2		(B) Tai chi (n.r., 1 month, n = 30)				
	(n.r.)						
Weatherley-Jones (2004) [17]	103/92	Oxford	(A) Homeopathy (6 months, n = 47)	(B) Placebo(6 months, n = 45)	1) MFI	1) NS except general fatigue, P = 0.04	n.r.
	n.r.				2) FIS	2) NS	
	Over 18 years				3) FLP	3) P = 0.04	
	(n.r)						
Awdry (1996) [18]	64/61	Oxford	(A) Homeopathy (1 year, n = 30)	(B) Placebo (n = 31)	1) Daily graphs	1-2) No between	n.r.
	18/43				2) Symptoms score	group analysis	
	<65						
	(n.r.)						
Hartz Hartz (2004) [19]	96/76	Fukuda	(A) Ginseng (Siberian, 2 months, n = 40	(B) Placebo (16 weeks, 2 month, n = 36)	RVI	NS	n.r.
	n.r.						
	21~65						
	(n.r)						
Forsyth (1999) [20]	26	CDC	(A) NADH (10 mg, once daily for 4 weeks, n = 26, cross over design)	(B) Placebo(n = 26)	Symptom score	P < 0.05	Overly stimulated, mild loss of appetite, heartburn, increased incidence of gas and an odd taste and dryness (1)
	(9/17)						
	26-57						
	-39.6						
Santaella (2004) [21]	31	Fukuda	(A) NADH or nutritional	(B) Psychological therapy (24 months, n = 11)	Symptom score (month 3)	P < 0.001	n.r.
	n.r.		supplements (5-10 mg, 24 months, n = 20)				
	22~54						
	(n.r)						
Brouwers (2002) [22]	53	CDC	(A) General nutritional supplement (twice a day for 10 weeks, n = 27)	(B) Placebo(n = 26)	1) Fatigue severity	1)-3) NS	n.r.
	(16/37)				2) Functional impairment		
	n.r.				3) Physical activity levels		
	-39.3						
De Becker (2001) [23]	90	Fukuda or Holmes	(A) Acclydine (250 mg, 4 times daily for 1^st ^4 weeks and 250 mg, twice daily for 2^nd ^4 weeks, n = 45)	(B) Placebo (n = 45)	CGI	No between group analysis	n.r.
	n.r.						
	n.r.						
	(n.r.)						
The (2007) [24]	57	CDC	(A) Food supplement (Acclydine 1000 mg to 250 mg for 14 weeks, n = 30)	(B) Placebo (n = 27)	1) Fatigue severity	1)-4) NS	Mild nausea (1), exacerbation of CFS symptoms (1) and irritable bowel symptoms (1)
	(20/37)				2) Function impairment		
	n.r.				3) Activity level		
	(n.r.)				4) Daily fatigue level		
McDertmott (2006) [25]	71	CDC	(A) Food supplement (Biobran 2 mg three times per day for 8 weeks, n = 37)	(B) Placebo(n = 34)	1) Fatigue scale	1) NS	
	(20/51)				2) QOL	2) NS	
	n.r.				(WHOQOL_BREF)		
	(n.r.)						
Stewart (1987) [26]	12	Not stated	(A) Supplements (subjects given supplements for 3 weeks, after first 3 weeks cross-over treatment arms for further 3 weeks, 2 multi-digestive enzymes, crossover design n = 12)	(B) Placebo(n = 12)	1) Fatigue	1)-2) No between group analysis	n.r.
	n.r.				2) Bowel movements		
	n.r.						
	(n.r.)						
Rothschild (2002) [27]	70/68	CDC	(A) Supplement (mushrooms plus aloe vera and cat's claw, processed and fermented, 3 caplets taken 3 times daily before meals, n = 33)	(B) Placebo (n = 35)	Symptoms	No between	n.r.
	20/50					group analysis	
	n.r.						
	(n.r.)						
Vermeulen (2004) [28]	90	CDC	(A) Acetyl-L-carnitine (2 g daily for 24 weeks, n = 30)	(B) Propionyl-L-carnitine (n = 30)	1) Fatigue scale	No between group analysis	n.r.
	(21/69)			(C) A plus B (n = 30)	2) Pain		
	n.r.				3) Attention/concentration		
	(n.r.)						
Behan (1990) [29]	63	Not stated	(A) Fatty acids((Efamol Marine, 8 capsules per day, n = 39)	(B) Placebo (n = 24)	1) Symptom measure	1) P < 0.001	n.r.
	27/36				2) General health	2) P < 0.0001	
	21-63					3) n.r.	
	-40						
Warren (1999) [30]	50	Oxford	(A) Essential fatty acids (Efamol Marine 1000 mg 4 times a day for 3 months, n = 25)	(B) Placebo (n = 25)	1) Physical symptom	1) NS	n.r.
	(21/29)				2) Depression (Beck Depression Inventory)	2) NS	
	18-59						
	-35.7						
Kaslow (1989) [31]	14	CDC	(A) Liver extract-folic acid-cyanocobalamin (intramuscular injection 2 ml for 1-week, n = 14, crossover design)	(B)Placebo (n = 14)	Functional status questionnaire	NS	None
	(3/11)						
	30-48						
	(n.r.)						
Ockerman (2000) [32]	22	CDC	(A)Antioxidant treatment (pollen and pistil 7 tablets per day for 3 months, n = 22, cross-over design)	(B) Placebo (3 month, n = 22)	1) Total well-being	1) No between group analysis	n.r.
	(3/19)				2) Clinical symptoms	2) No between group analysis	
	27-70						
	-50						
Cox (1991) [33]	34/31	other	(A) Vitamin and minerals (Magnesium, 6 weeks, n = 14)	(B) Placebo (6 weeks, n = 17)	Mean Nottingham	P = 0.001	n.r.
	23-Nov				health profile score		
	18~56						
	(n.r.)						
Tiev (1999)	326	Not stated	(A) Sulbutiamine (400 mg	(C) Placebo (n = 109)	1) MFI	1) Sleep disturbance	(A):9,(B):6,(C):12 diarrhoea, cystitis, bronchitis, arthritic pain, back pain, asthma, abdominal pain, insomnia, constipation, gastroenteritis, diffuse pain, sinusitis, headache, renal coli, vertigo, pharyngitis, tracheitis.
			daily, n = 106)		2) Clinical global impression	(p = 0.03)	
			(B) Sulbutiamine (600 mg		3) Baecke's measure of activity	Pain (p = 0.044)	
			daily, n = 111)		4) Illness severity	2) NS	
						3) NS	
Willams (2002) [35]	30	Oxford	(A) Melatonin (5 mg in the	(B) phototherapy (2500 Lux for 1	1) VAS for symptom severity; SF-36	1) Sleep disturbance(p = 0.03);Pain (p = 0.044)	n.r.
	13/17		evening, 12 week, n = 30)	hour in the morning, 12 weeks, n = 30)	2) Mental fatigue	2) NS	
	n.r.				3) HADS	3) NS	
	-44.5						

CDC: Centers for Disease Control and Prevention; CESD: Center for Epidemiological Studies Depression Scale; CFS: Chronic Fatigue Syndrome; CGI: Clinician's Global Impression Scale; FIS: Fatigue Impact Scale; FLP: Functional Limitations Profile; HADS: Hospital Anxiety and Depression Scale; MBSR: Mindfulness-Based Stress Reduction; MFI: Multidimensional Fatigue Inventory; n.r.: not reported; NS: Not significant; RVI: Rand Vitality Index; NADH: Nicotinamide adenine dinucleotide; QoL: Quality of life; SF-36: Short Form 36; VAS: Visual Analogue Scale; WHOQOL-BREF: WHO Quality of Life-BREF.

**Table 2:** Risk of bias of included RCTs*

Study	Random sequence generation	Allocation concealment	Patient blinding	Assessor blinding	Reporting drop-out or withdrawal^†^	Intention-to-treat analysis^†^	Selective outcome reporting	Other potential bias
Clollinge (1998) [10]	Low	Low	High	Low	Low	High	High	Unclear
Dybwad (2007) [11]	Low	Low	High	Low	Low	Unclear	Low	Low
Surawy (2005) [12]	Unclear	Unclear	High	Unclear	High	Unclear	Unclear	Unclear
Walach (2008) [13]	Low	Low	Low	Low	Low	High	Low	Low
Field (1997) [14]	Low	Unclear	High	High	Unclear	Unclear	Unclear	High
Wang (2009) [15]	Unclear	Unclear	Low	Low	High	Unclear	Low	Unclear
Liu (2010) [16]	Unclear	Unclear	High	Unclear	Unclear	Unclear	Unclear	Unclear
Weatherley-Jones (2004) [17]	Low	Low	Low	Low	Low	Low	Low	Low
Awdry (1996) [18]	Unclear	Unclear	Low	Low	High	High	Low	Unclear
Hartz (2004) [19]	Low	Low	Low	Low	Low	Low	Low	Low
Forsyth (1999) [20]	Unclear	Unclear	Low	Unclear	Unclear	High	Unclear	Unclear
Santaella (2004) [21]	Unclear	Unclear	High	High	High	High	Low	Unclear
Brouwers (2002) [22]	Unclear	High	Low	Low	Low	Low	Unclear	Low
De Becker (2001) [23]	Unclear	Low	Low	Unclear	Unclear	Unclear	Unclear	Low
The (2007) [24]	Low	Low	Low	Low	Low	Low	Unclear	Low
McDertmott (2006) [25]	Low	Low	Low	Low	Low	High	Low	Low
Stewart (1987) [26]	Unclear	Unclear	High	High	Low	High	High	Unclear
Rothschild (2002) [27]	Unclear	Unclear	Low	Low	High	High	Unclear	Unclear
Vermeulen (2004) [28]	Low	Low	Low	Low	Low	Low	Low	Low
Behan (1990) [29]	Low	Low	High	High	Low	Unclear	High	Unclear
Warren (1999) [30]	Low	Low	Low	Low	Low	Low	Low	Unclear
Kaslow (1989) [31]	Unclear	Low	Low	Low	Low	Unclear	Unclear	Low
Ockerman (2000) [32]	Unclear	Low	Low	Low	Low	Unclear	Unclear	Low
Cox (1991) [33]	Unclear	Unclear	Low	Low	Unclear	High	Low	Unclear
Tiev (1999) [34]	Unclear	Unclear	Unclear	Unclear	Low	Unclear	Unclear	Unclear
Willams (2002) [35]	Unclear	Unclear	Unclear	Unclear	Low	Unclear	Unclear	Unclear

*, Domains of quality assessment based on Cochrane tools for assessing risk of bias.

^†^, Two domains referring to 'incomplete outcome data' in the Cochrane tools for assessing risk of bias.

^‡^, This study had a baseline imbalance in the subjective outcome values.

Abbreviations; Low (low risk of bias);/High (high risk of bias); Unclear (uncertain risk of bias).

### Risk of bias

The risk of bias in the studies was variable. Eleven RCTs had an adequate method for random sequence generation [10,11,13,15,17,19,24,25,28,29,33], whereas the remaining 15 RCTs did not [12,14,16,18,20-23,26,27,30-32,34,35]. Allocation concealments were adequately performed in 13 RCTs [10,11,13,17,19,24,25,29-33]. Patient and assessor blinding was reported in 16 of the RCTs [17-20,22-27,29-34], whereas two RCTs employed assessor blinding only [10,11]. Reasons for dropouts and withdrawals were fully described in 17 trials [10,11,13,17,19,22-26,28-30,32-35]. With respect to the intention-to-treat (ITT) analysis, 11 RCTs did not report the basis of the analysis [11,12,14-16,28,30-32,34,35], and 9 were analysed on a per-protocol basis [10,13,18,20,21,23,25-27]. The remaining 6 studies employed the ITT method [17,19,22,24,29,33]. Eleven RCTs had a low risk of bias in selective outcome reporting [11,13,14,17-19,21,23,25,29,33], and the others had a high risk of such bias.

#### Mind-body and energy medicine

Two RCTs compared qigong plus meditation with no treatment [10,11]. Both studies reported beneficial effects of qigong with meditation on fatigue. One RCT tested Mindfulness Based Stress Reduction (MBSR) as compared with a wait-list control and found significant effects of the treatment on anxiety [12]. The other RCT compared distant healing with a four-armed partial blinding, placebo-controlled design and did not show a significant effect on mental or physical components of quality of life [13].

#### Massage

Two RCTs tested massage compared with Sham TENS or another type of massage [14,15]. One RCT demonstrated the beneficial effects of massage on several symptoms of CFS, including depression, fatigue, pain and insomnia [14]. The other RCT compared a special type of massage (Intelligent-turtle) with general massage and reported some effect of this type of massage on physical symptoms [15].

#### Tuina and tai chi

One RCT tested tuina and tai chi as compared to fluoxetine [16]. The tuina group had more symptom reduction than the fluoxetine group fluoxetine, but there were no significant differences between tuina and tai chi or tai chi and fluoxetine after 1 month of treatment. The effective rate was decided by the practitioner and was based on symptom improvement, which was not described in detail.

#### Homeopathy

Two RCTs compared homeopathy with placebo [17,18]. One RCT showed that homeopathy improved fatigue and function [17]. The other RCT reported the beneficial effects of homeopathy on symptom improvement [18].

#### Ginseng

One RCT tested Siberian ginseng and failed to show the effectiveness of ginseng on the Rand Vitality Index [19].

#### Supplements

Two RCTs compared NADH with placebo or psychological therapy [20,21]. One RCT showed statistically significant effects of NADH (10 mg) on symptom scores when compared with placebo after 1 month of treatment [20]. The other RCT also reported the positive effects of NADH (from 5 to 10 mg) when compared with psychological therapy (not reported in details) after 3 months [21].

Six RCTs compared several types of general food supplements with a placebo control [22-27]. Five of these RCTs failed to show significant effects of dietary supplements on symptoms of CFS when compared with placebo. One RCT compared acclydine with placebo and showed the beneficial effects of acclydine on clinical improvement at weeks 4 and 8 [23].

One RCT tested Acetyl-L-carnitine as compared with Propionyl-carnitine and combined both therapies [28]. The results showed the beneficial effects of each therapy on fatigue, pain and attention/concentration.

Two RCTs compared essential fatty acids (Efamol Marine-evening primrose oil) with placebo [29,30]. One RCT showed the possible efficacy of essential fatty acids on symptoms and general heath [29], whereas the other RCT failed to show an impact of this therapy on physical symptoms and depression when compared to placebo [30].

One RCT compared liver extract-folic acid-cyanocobalamin with placebo and failed to show an effect for the treatment [31]. The second RCT compared antioxidant treatment with placebo and reported beneficial effects in the treatment group; however, there were no reports on intergroup differences [32]. The third RCT compared a magnesium supplement with placebo and found beneficial effects of magnesium on patients' symptom profiles [33]. A large, double-blind RCT of patients with CFS investigated the effect of isobutyryl-thiamine disulphide. No improvements were observed when compared with placebo [34]. When melatonin was compared in an RCT with phototherapy, neither intervention generated beneficial effects [34].

#### Adverse events

Five of the 26 included studies reported no adverse events or a slight occurrence of them [16,20,24,31,34], whereas the remaining 20 studies lacked descriptions regarding the occurrence of adverse events.

## Discussion

Our analysis shows that a range of CAM studies have been conducted to determine which therapies might ameliorate CFS symptoms. There is insufficient evidence to conclusively determine efficacy. Studies of qigong, massage and tuina have demonstrated positive effects; however, the nature of the control group and the quality of the studies prevent us from concluding that those CAM therapies are effective for CFS. Compared with placebo, homeopathy also had insufficient evidence of symptom improvement in CFS. Seventeen studies tested supplements for CFS. Most of the supplements failed to show favourable effects for CFS, with the exception of NADH and magnesium. However, the total number of RCTs and the total sample size were too small to draw firm conclusions.

Our review aimed to update and complete the evidence of CAM treatments for symptom relief in patients with CFS. Compared to a previous review [7], we identified 3 new types of CAM and 9 new RCTs and successfully updated the evidence for these therapies in CFS. The results of our review are similar to that of the previous review [7], which also expressed concern regarding the poor methodological quality of the included primary studies [7]. Another two published reviews concerning traditional Chinese medicine and herbal medicines were unable to find appropriate studies to review [36,37].

Most of the included trials had a high risk of bias in many domains. Low quality trials are more likely to overestimate effect size [38]. This is also true for trials with inadequate blinding and inadequate allocation concealment, as such trials are more subject to selection bias and are likely to generate exaggerated treatment effects [38,39]. Several trials used an inadequate method for sequence generation. Because inadequate sequence generation in randomisation studies also tends to yield a larger estimate of treatment effects, this is another source of potential bias.

One argument for using CAM for the management of CFS might be that it causes fewer adverse effects than drug treatment. Only five RCTs [16,20,24,31,34] assessed the adverse effects of CAM treatment, while 21 RCTs did not. No severe adverse effects of CAM were noted. However, adverse effects should be assessed in future CAM trials. This is an important factor for patients, as CAM treatments are generally offered outside of the official health care system.

Our review has a number of important limitations. Although strong efforts were made to retrieve all RCTs on the subject, we cannot be absolutely certain that we succeeded. Moreover, selective publishing and reporting are other major causes for bias, which must be considered [40,41]. It is conceivable that several negative RCTs remain unpublished, thus distorting the overall picture [40,42]. Further limitations include the paucity and often suboptimal methodological quality of the primary data. Together, these factors limit the conclusiveness of this systematic review considerably.

## Conclusions

The results of our systematic review provide limited evidence for the effectiveness of CAM in treating patients with CFS. However, the total number of RCTs included in the analysis, the total sample size and their risk of bias were quite high in several domains; thus, drawing firm conclusions concerning the effectiveness of CAM therapies remains difficult. Further rigorous RCTs that can overcome the many limitations of the current literature are warranted.

## Competing interests

The authors declare that they have no competing interests.

## Authors' contributions

TA obtained funding for the study, conceived and participated in its design and coordination and helped to draft the manuscript. MSL conceived and participated in its design. MSL and TYC searched databases, extracted and assessed studies. They also helped to draft the manuscript. HC participated in the study design and helped to draft the manuscript. JL conceived the study, and participated in its design and helped to draft the manuscript. All authors read and approved the final manuscript.

## Pre-publication history

The pre-publication history for this paper can be accessed here:

http://www.biomedcentral.com/1472-6882/11/87/prepub

## Supplementary Material

Additional file 1 Search Strategy Click here for file

## References

[B1] Jason LA Evans M Brown M Porter N What is fatigue? Pathological and nonpathological fatigue PM R 2010 2 5 327 331 20656613 10.1016/j.pmrj.2010.03.028

[B2] Rusmevichientong A Chow SA Biology and pathophysiology of the new human retrovirus XMRV and its association with human disease Immunol Res 2010 48 1-3 27 39 10.1007/s12026-010-8165-y 20717743

[B3] Van Houdenhove B Kempke S Luyten P Psychiatric aspects of chronic fatigue syndrome and fibromyalgia Curr Psychiatry Rep 2010 12 3 208 214 10.1007/s11920-010-0105-y 20425282

[B4] Bagnall AM Whiting P Richardson R Sowden AJ Interventions for the treatment and management of chronic fatigue syndrome/myalgic encephalomyelitis Qual Saf Health Care 2002 11 3 284 288 10.1136/qhc.11.3.284 12486997 PMC1743629

[B5] Chambers D Bagnall AM Hempel S Forbes C Interventions for the treatment, management and rehabilitation of patients with chronic fatigue syndrome/myalgic encephalomyelitis: an updated systematic review J R Soc Med 2006 99 10 506 520 10.1258/jrsm.99.10.506 17021301 PMC1592057

[B6] Whiting P Bagnall AM Sowden AJ Cornell JE Mulrow CD Ramirez G Interventions for the treatment and management of chronic fatigue syndrome: a systematic review JAMA: the journal of the American Medical Association 2001 286 11 1360 1368 10.1001/jama.286.11.1360 11560542

[B7] Porter NS Jason LA Boulton A Bothne N Coleman B Alternative medical interventions used in the treatment and management of myalgic encephalomyelitis/chronic fatigue syndrome and fibromyalgia J Altern Complement Med 2010 16 3 235 249 10.1089/acm.2008.0376 20192908

[B8] Higgins JPT Altman DG Sterne JAC Higgins JPT, Green S Chapter 8: Assessing risk of bias in included studies Cochrane Handbook for Systematic Reviews of Interventions Version 510 (updated March 2011) 2011 The Cochrane Collaboration http://www.cochrane-handbook.org

[B9] Zhang W Liu Z Wu T Peng W Acupuncture for chronic fatigue syndrome Cochrane DB Syst Rev 2006 CD006010

[B10] Collinge W Yarnold PR Raskin E Use of mind-body selfhealing practice predicts positive health transition in chronic fatigue syndrome: a controlled study Subtle Energies Energy 1998 9 171 190

[B11] Dybwad MH Frøslie KF Stanghelle JK Work capacity, fatigue and health related quality of life in patients with myalgic encephalopathy or chronic fatigue syndrome, before and after qigong Therapy, a randomized controlled study Nesoddtangen, Norway: Sunnaas Rehabilitation Hospital 2007 http://old.sunnaas.no/stream_file.asp?iEntityId=7623

[B12] Surawy C Roberts J Silver A The effect of mindfulness training on mood and measures of fatigue, activity, and quality of life in patients with chronic fatigue syndrome on a hospital waiting list: a series of exploratory studies Behav Cogn Psychother 2005 33 103 109 10.1017/S135246580400181X

[B13] Walach H Bosch H Lewith G Naumann J Schwarzer B Falk S Kohls N Haraldsson E Wiesendanger H Nordmann A Effectiveness of distant healing for patients with chronic fatigue syndrome: a randomised controlled partially blinded trial (EUHEALS) Psychother Psychosom 2008 77 3 158 166 10.1159/000116609 18277062

[B14] Field TM Sunshine W Hernandez-Reif M Quintino O Schanberg S Kuhn C Burman I Massage therapy effects on depression and somatic symptoms in chronic fatigue syndrome J Chronic Fatigue Syndr 1997 3 43 51

[B15] Wang JH Chai TQ Lin GH Luo L Effects of the intelligent-turtle massage on the physical symptoms and immune functions in patients with chronic fatigue syndrome J Tradit Chin Med 2009 29 1 24 28 10.1016/S0254-6272(09)60026-1 19514184

[B16] Liu CZ Lei B Effect of Tuina on oxygen free radicals metabolism in patients with chronic fatigue syndrome Zhongguo Zhen Jiu 2010 30 11 946 948 21246855

[B17] Weatherley-Jones E Nicholl JP Thomas KJ Parry GJ McKendrick MW Green ST Stanley PJ Lynch SP A randomised, controlled, triple-blind trial of the efficacy of homeopathic treatment for chronic fatigue syndrome J Psychosom Res 2004 56 2 189 197 10.1016/S0022-3999(03)00377-5 15016577

[B18] Awdry R Homeopathy may help ME Int J Alternat Complement Med 1996 14 12 16

[B19] Hartz AJ Bentler S Noyes R Hoehns J Logemann C Sinift S Butani Y Wang W Brake K Ernst M Randomized controlled trial of Siberian ginseng for chronic fatigue Psychol Med 2004 34 1 51 61 14971626 10.1017/s0033291703008791

[B20] Forsyth LM Preuss HG MacDowell AL Chiazze L Jr Birkmayer GD Bellanti JA Therapeutic effects of oral NADH on the symptoms of patients with chronic fatigue syndrome Ann Allergy Asthma Immunol 1999 82 2 185 191 10.1016/S1081-1206(10)62595-1 10071523

[B21] Santaella ML Font I Disdier OM Comparison of oral nicotinamide adenine dinucleotide (NADH) versus conventional therapy for chronic fatigue syndrome P R Health Sci J 2004 23 2 89 93 15377055

[B22] Brouwers FM Van Der Werf S Bleijenberg G Van Der Zee L Van Der Meer JW The effect of a polynutrient supplement on fatigue and physical activity of patients with chronic fatigue syndrome: a double-blind randomized controlled trial QJM 2002 95 10 677 683 10.1093/qjmed/95.10.677 12324640

[B23] De Becker P Nijs J Van HE McGregor N De MK A double-blind, placebo-controlled study of acclydine in combination with amino acids in patients with chronic fatigue syndrome AHMF Proceedings "Myalgic Encephalopathy/Chronic Fatigue Syndrome The Medical Practitioners' Challenge in 2001" 2001 http://www.prohealth.com/library/showarticle.cfm?libid=8547

[B24] The GKH Bleijenberg G van der Meer JWM The Effect of Acclydine in Chronic Fatigue Syndrome: A Randomized Controlled Trial PLOS Clin Trial 2007 2 5 e19 10.1371/journal.pctr.0020019 PMC1876596 17525791

[B25] McDermott C Richards SC Thomas PW Montgomery J Lewith G A placebo-controlled, double-blind, randomized controlled trial of a natural killer cell stimulant (BioBran MGN-3) in chronic fatigue syndrome QJM 2006 99 7 461 468 10.1093/qjmed/hcl063 16809351

[B26] Stewart W Rowse C Supplements help ME says Kiwi study J Altern Complement Med 1987 5 19 20

[B27] Rothschild PR Huertas JG Ambulatory naturopathic treatment of chronic fatigue immune deficiency syndrome (CFIDS) with RM-10 caplets Progress in Nutrition 2002 4 77 96

[B28] Vermeulen RC Scholte HR Exploratory open label, randomized study of acetyl- and propionylcarnitine in chronic fatigue syndrome Psychosom Med 2004 66 2 276 282 10.1097/01.psy.0000116249.60477.e9 15039515

[B29] Behan PO Behan WM Horrobin D Effect of high doses of essential fatty acids on the postviral fatigue syndrome Acta Neurol Scand 1990 82 3 209 216 10.1111/j.1600-0404.1990.tb04490.x 2270749

[B30] Warren G McKendrick M Peet M The role of essential fatty acids in chronic fatigue syndrome. A case-controlled study of red-cell membrane essential fatty acids (EFA) and a placebo-controlled treatment study with high dose of EFA Acta Neurol Scand 1999 99 2 112 116 10.1111/j.1600-0404.1999.tb00667.x 10071170

[B31] Kaslow JE Rucker L Onishi R Liver extract-folic acid-cyanocobalamin vs placebo for chronic fatigue syndrome Arch Intern Med 1989 149 11 2501 2503 10.1001/archinte.149.11.2501 2684076

[B32] Ockerman PA Antioxidant treatment of chronic fatigue syndrome Clinical Practice of Alternative Medicine 2000 1 88 91

[B33] Cox IM Campbell MJ Dowson D Red blood cell magnesium and chronic fatigue syndrome Lancet 1991 337 8744 757 760 10.1016/0140-6736(91)91371-Z 1672392

[B34] Tiev KP Cabane J Imbert JC [Treatment of chronic postinfectious fatigue: randomized double-blind study of two doses of sulbutiamine (400-600 mg/day) versus placebo] Rev Med Interne 1999 20 10 912 918 10.1016/S0248-8663(00)80096-X 10573727

[B35] Williams G Waterhouse J Mugarza J Minors D Hayden K Therapy of circadian rhythm disorders in chronic fatigue syndrome: no symptomatic improvement with melatonin or phototherapy European Journal of Clinical Investigation 2002 32 11 831 837 10.1046/j.1365-2362.2002.01058.x 12423324

[B36] Adams D Wu T Yang X Tai S Vohra S Traditional Chinese medicinal herbs for the treatment of idiopathic chronic fatigue and chronic fatigue syndrome Cochrane Database Syst Rev 2009 4 CD006348 10.1002/14651858.CD006348.pub2 19821361

[B37] Chen R Moriya J Yamakawa JI Takahashi T Kanda T Traditional Chinese Medicine for Chronic Fatigue Syndrome Evid Based Complement Alternat Med 2008 10.1093/ecam/nen017 PMC2816380 18955323

[B38] Schulz KF Chalmers I Hayes RJ Altman DG Empirical evidence of bias. Dimensions of methodological quality associated with estimates of treatment effects in controlled trials JAMA 1995 273 5 408 412 10.1001/jama.273.5.408 7823387

[B39] Day SJ Altman DG Statistics notes: blinding in clinical trials and other studies BMJ 2000 321 7259 504 10.1136/bmj.321.7259.504 10948038 PMC1118396

[B40] Ernst E Pittler MH Alternative therapy bias Nature 1997 385 6616 480 9020351 10.1038/385480c0

[B41] Pittler MH Abbot NC Harkness EF Ernst E Location bias in controlled clinical trials of complementary/alternative therapies J Clin Epidemiol 2000 53 5 485 489 10.1016/S0895-4356(99)00220-6 10812320

[B42] Rothstein HR Sutton AJ Borenstein M Rothstein HR, Sutton AJ, Borenstein M Publication bias in meta-analysis Publication bias in meta-analysis 2005 Chichester West Sussex: Wiley

