# Supplementary material for: Complementary and alternative medicine for patients with chronic fatigue syndrome: A systematic review
Source: BMC Complement Altern Med. 2011 Oct 7;11:87. doi: 10.1186/1472-6882-11-87 (PMC3201900; doi:10.1186/1472-6882-11-87)
Supplement: Additional file 1 — Search Strategy [file 1472-6882-11-87-S1.DOC]

**Additional file 1. Search strategy**

**MEDLINE (OvidSP)**

1 chronic fatigue syndrome or CFS/
2 myalgic encephalomyelitis or ME/
3 or/1-2
4 complementary and alternative medicine/
5 complementary and alternative medicine.tw.
6 mind body medicine/
7 mind body medicine.tw.
8 qigong/
9 qigong.tw.
10 aromatherapy/
11 aromatherapy.tw.
12 reiki/
13 reiki.tw.
14 touch/
15 touch.tw.
16 herb/
17 herb.tw.
18 yoga/
19 yoga.tw.
20 tuina/
21 tuina.tw.
22 massage/
23 massage.tw.
24 chiropractic/
25 chiropractic.tw.
26 hypnosis/
27 hypnosis.tw.
28 homeopathy/
29 homeopathy.tw.
30 natural supplement/
31 natural supplement.tw.
32 dietary supplement/
33 dietary supplement.tw.
34 or/4-33
35 3 and 34

**EMBASE**

#1 ‘chronic fatigue syndrome’/exp OR ‘chronic fatigue syndrome’
#2 ‘myalgic encephalomyelitis’/exp OR ‘myalgic encephalomyelitis’
#3 #1 OR #2
#4 ‘complementary and alternative medicine’/exp OR ‘complementary and alternative medicine’
#5 ‘mind body medicine’/exp OR ‘mind body medicine’.
#6 ‘qigong’/exp OR qigong
#7 ‘aromatherapy’/exp OR aromatherapy
#8 ‘reiki’/exp OR reiki
#9 ‘touch’/exp OR ‘touch’
#10 ‘herb’/exp OR herb
#11 ‘yoga’/exp OR yoga
#12 ‘tuina’/exp OR tuina
#13 ‘massage’/exp OR massage
#14 ‘chiropractic’/exp OR chiropractic
#15 ‘hypnosis’/exp OR hypnosis
#16 ‘homeopathy’/exp OR homeopathy
#17 ‘natural supplement’/exp OR ‘natural supplement’
#18 ‘dietary supplement’/ OR ‘dietary supplement’
#19 #4 OR #5 OR #6 OR #7 OR #8 OR #9 OR #10 OR #11 OR #12 OR #13 OR #14 OR #15 OR #16 OR #17 OR #18
#20 #3 AND #19
